# Quacks and Their Methods

**Published:** 1883-04

**Authors:** 


### QUACKS AND THEIR METHODS.

IT is not difficult to understand why even intelligent people are sometimes deceived by the flaming advertisements of quacks, and are induced to try their nostrums. Many persons suffer from disease, without being able to find relief through the advice of regular physicians, and are disposed to catch at anything that promises a cure.

Then there are thousands of imaginary invalids, who think that every transient ache means mischief; they invest freely in patent medicines, and no doubt often believe that they derive benefit from them.

There is also a large class of people who seem to think that good health can be maintained only by constant dosing.

But one of the most plausible devices of charlatans to give themselves and their medicines respectability is the pretence of being connected with some prominent publication. There are quite a number of quacks, some with fictitious names, who claim that the JOURNAL OF HEALTH indorses their remedies, while others pretend that they are contributors to the JOURNAL; and there are at least two who profess to be associate editors with us.

The business career of one of our *imaginary* associate editors was brought to a sudden close by the Post-Master General, who directed that his name should be printed in the list of FRAUDS, in the P. O. Directory. The other—who was formerly a partner of the first—still announces himself as an associate editor of HALL'S JOURNAL OF HEALTH, for the purpose of pushing the sale of some sort of a *cure-all pad.* We should not take the trouble of referring to this matter, were it not that numbers of our subscribers—some of them esteemed personal friends—have written to us inquiring about the merits of this *pad.* We therefore take occasion to say, that we never had the *pleasure* of the acquaintance of either of our *pretended* editorial associates, and have never seen the *pads* or other "gimcracks" that we are said to indorse; moreover, we indorse no secret remedies, as we believe every man has as much right to know what medicine he takes as to know what is the character of the food he eats.

We are particularly careful not to admit that class of advertisements in the JOURNAL. We take occasion to answer in advance inquiries that are often made of us as to the merits of the many things we do advertise, by saying that our advertisers are all men of the highest standing as business men, and that implicit reliance can be placed on statements made in reference to their goods.

